# Highly‐Tunable Intrinsic Room‐Temperature Ferromagnetism in 2D van der Waals Semiconductor Cr*_x_* Ga_1−_ *_x_* Te

**DOI:** 10.1002/advs.202103173

**Published:** 2021-10-27

**Authors:** Gaojie Zhang, Hao Wu, Liang Zhang, Shanfei Zhang, Li Yang, Pengfei Gao, Xiaokun Wen, Wen Jin, Fei Guo, Yuanmiao Xie, Hongda Li, Boran Tao, Wenfeng Zhang, Haixin Chang

**Affiliations:** ^1^ Center for Joining and Electronic Packaging State Key Laboratory of Material Processing and Die & Mold Technology School of Materials Science and Engineering Huazhong University of Science and Technology Wuhan 430074 China; ^2^ Institute for Quantum Science and Engineering Huazhong University of Science and Technology Wuhan 430074 China; ^3^ Center for Materials Science and Engineering School of Electrical and Information Engineering Guangxi University of Science and Technology Liuzhou 545006 China

**Keywords:** Cr*_x_* Ga_1−_ *_x_* Te, room‐temperature ferromagnetism, semiconductors, van der Waals

## Abstract

The combination of semiconductivity and tunable ferromagnetism is pivotal for electrical control of ferromagnetism and next‐generation low‐power spintronic devices. However, Curie temperatures (*T*_C_) for most traditional intrinsic ferromagnetic semiconductors (≤200 K) and recently discovered two‐dimensional (2D) ones (<70 K) are far below room temperature. 2D van der Waals (vdW) semiconductors with intrinsic room‐temperature ferromagnetism remain elusive considering the unfavored 2D long‐range ferromagnetic order indicated by Mermin–Wagner theorem. Here, vdW semiconductor Cr*_x_* Ga_1−_ *_x_* Te crystals exhibiting highly tunable above‐room‐temperature ferromagnetism with bandgap 1.62–1.66 eV are reported. The saturation magnetic moment (*M*_sat_) of Cr*_x_* Ga_1−_ *_x_* Te crystals can be effectively regulated up to ≈5.4 times by tuning Cr content and ≈75.9 times by changing the thickness. vdW Cr*_x_* Ga_1−_ *_x_* Te ultrathin semiconductor crystals show robust room‐temperature ferromagnetism with the 2D quantum confinement effect, enabling *T*_C_ 314.9–329 K for nanosheets, record‐high for intrinsic vdW 2D ferromagnetic semiconductors. This work opens an avenue to room‐temperature 2D vdW ferromagnetic semiconductor for 2D electronic and spintronic devices.

## Graphical abstract

2D van der Waals (vdW) semiconductor Cr*_x_* Ga_1−_ *_x_* Te with highly tunable intrinsic room‐temperature ferromagnetism is realized and shows robust room‐temperature ferromagnetism with the 2D quantum confinement effect, enabling *T*_C_ record‐high 314.9–329 K for 2D semiconductors. The *M*_sat_ of vdW Cr*_x_* Ga_1−_ *_x_* Te crystals can be effectively tuned up to ≈5.4 times by controlling Cr concentration and up to ≈75.9 times by changing the thickness.

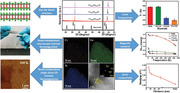

## 1 Introduction

Ferromagnetic semiconductors are fundamental for next‐generation spintronics because of their greater carrier regulation potential than ferromagnetic metals.^[^ ^1^ ^]^ The realization of intrinsic ferromagnetic semiconductors will promote the development of electrical manipulation of magnetic states^[^ ^2^, ^3^ ^]^ and semiconducting spintronic devices.^[^ ^4^, ^5^ ^]^ The most significant requirement is semiconducting ferromagnetic semiconductor with above‐room‐temperature Curie temperature (*T*_C_) and tunable ferromagnetic properties. However, despite considerable efforts, most traditional intrinsic ferromagnetic semiconductors have *T*_C_ still far below room temperature (e.g., ≈200 K for MnGaAs).^[^ ^6^, ^7^, ^8^ ^]^ In addition, recently discovered intrinsic van der Waals (vdW) ferromagnetic semiconductors CrX_3_ (X = Br and I),^[^ ^9^, ^10^ ^]^ Cr_2_X_2_Te_6_ (X = Si and Ge),^[^ ^11^, ^12^ ^]^ and VI_3_^[^ ^13^, ^14^ ^]^ inspire the research of intrinsic 2D ferromagnetism and various functional spintronic devices such as electron tunneling probing of ferromagnetism, spin quantum sensors, giant tunneling magnetoresistance, and spin tunnel field‐effect transistors.^[^ ^15^, ^16^, ^17^, ^18^, ^19^ ^]^ However, similarly, their extremely low *T*_C_ (mostly 10–68 K)^[^ ^9^, ^10^, ^11^, ^12^, ^13^, ^14^ ^]^ make the resulted devices and physical properties to be operated only at conditions far below room temperature.The realization of intrinsic room‐temperature ferromagnetism in the vdW semiconductors is pivotal for electric‐field control of 2D ferromagnetism and next‐generation low‐power spintronic devices.^[^ ^2^, ^3^, ^4^, ^5^ ^]^ Also, vdW semiconductors with intrinsic room‐temperature ferromagnetism remain elusive and are rarely observed for unfavored 2D long‐range ferromagnetic order as indicated by Mermin–Wagner theorem.^[^ ^20^ ^]^

Here, we successfully grow vdW semiconductor Cr*_x_* Ga_1−_ *_x_* Te single crystals by a two‐step KCl‐flux method and observe highly tunable, intrinsic room‐temperature ferromagnetism in both bulk Cr*_x_* Ga_1−_ *_x_* Te crystals and ultrathin Cr*_x_* Ga_1−_ *_x_* Te nanosheets. The *T*_C_ of intrinsic room‐temperature ferromagnetism in Cr*_x_* Ga_1−_ *_x_* Te nanosheets reaches record‐high 314.9–329 K for intrinsic vdW 2D ferromagnetic semiconductors. The *M*_sat_ of vdW Cr*_x_* Ga_1−_ *_x_* Te crystals can be effectively tuned up to ≈5.4 times by controlling the Cr concentration and up to ≈75.9 times by changing the thickness. Moreover, Cr*_x_* Ga_1−_ *_x_* Te possesses a moderate bandgap (1.62–1.66 eV), providing the greater potential for carrier control. This work realizes a highly tunable platform for intrinsic room‐temperature 2D ferromagnetism in vdW semiconductors and opens an avenue to room‐temperature vdW integrated spintronic devices.

## 2 Results and Discussion

**Figure 1:**
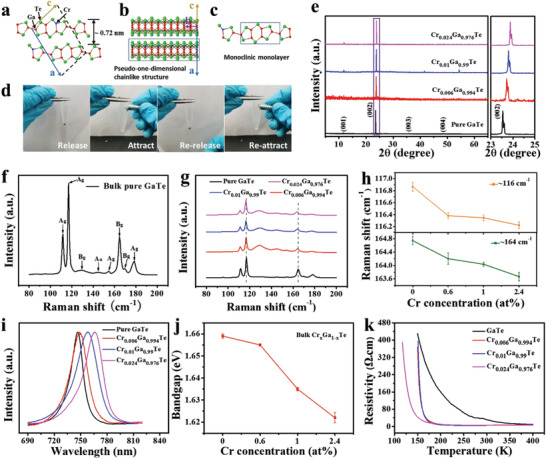
Crystal structure and characterizations of bulk vdW Cr*_x_* Ga_1−_ *_x_* Te crystals with different Cr concentrations. a,b) Front (a) and side view (b) for the crystal structure of monoclinic Cr*_x_* Ga_1−_ *_x_* Te. Red, green, and blue balls represent the Ga, Te, and Cr atoms, respectively. c) Front view of two vertical and one horizontal Te–Ga–Ga–Te units’ interconnection in one monolayer. The rectangles in (b) and (c) are the front and side view of pseudo‐1D chainlike structure. d) The Cr_0.024_Ga_0.976_Te crystals are immersed in NaCl solution, attracted by a ferromagnet and rereleased after attraction. e) XRD patterns of the Cr*_x_* Ga_1−_ *_x_* Te with different Cr concentrations. Right panel: enlarged view of the (002) peak. f) A typical Raman spectra of the GaTe. g) Raman spectra of the Cr*_x_* Ga_1−_ *_x_* Te with different Cr concentrations. h) The concentration‐dependent Raman shifts of ≈116 and ≈164 cm^−1^ peaks in (g). Error bars SD; *N* = 3. i) PL spectra of Cr*_x_* Ga_1−_ *_x_* Te with different Cr concentrations. j) The concentration‐dependent bandgap of Cr*_x_* Ga_1−_ *_x_* Te extracted from (i). Error bars SD; *N* = 3. k) Temperature‐dependent resistivity for Cr*_x_* Ga_1−_ *_x_* Te with different Cr concentrations.

Gallium telluride (GaTe) is a vdW direct‐bandgap semiconductor with the monoclinic structure of space group *C*2/*m* (*C*_2_ *_h_* ^3^) that has been widely studied in electronics and optoelectronics.^[^ ^21^, ^22^, ^23^ ^]^ With the introduction of Cr, a new vdW ferromagnetic semiconductor, Cr*_x_* Ga_1−_ *_x_* Te, is realized (**Figure** **1**a,b). Cr*_x_* Ga_1−_ *_x_* Te holds the monoclinic structure and has a pseudo‐1D feature with a chainlike structure arranged in the 2D layer plane. The pseudo‐1D chainlike structure in the crystal structure diagram refers to the long chain formed by the extension of the rectangle along the direction of *b*‐axis in Figure 1c, and is represented as the long strip crystal outline in the macroscopic crystal (purple arrows in Figure S1a–c in the Supporting Information).

In this work, GaTe and Cr*_x_* Ga_1−_ *_x_* Te single crystals were grown by a two‐step KCl‐flux method (see details in the Experimental Section), and the Cr concentration is determined by the energy dispersive spectrum (EDS) analysis (Table S1 and more discussions in Note S3 in the Supporting Information). The X‐ray fluorescence spectroscopy (XRF) with distinguished Cr was performed in a clean and smooth surface of an as‐exfoliated Cr_0.024_Ga_0.976_Te single crystal (Figure S1d, Supporting Information). It is worth to note that the Cr*_x_* Ga_1−_ *_x_* Te single crystals immersed in the NaCl solution can be attracted easily by a ferromagnet at room temperature (Figure 1d), implying the room‐temperature macroscopic ferromagnetism in Cr*_x_* Ga_1−_ *_x_* Te. Moreover, we did X‐ray diffraction (XRD) to compare the phase structure of bulk GaTe and Cr*_x_* Ga_1−_ *_x_* Te crystals. As shown in Figure 1e, all diffraction peaks are identified by the standard PDF card of monoclinic GaTe (PDF#44‐1127) and their locations are consistent with the previous reports of monoclinic GaTe.^[^ ^24^, ^25^ ^]^ The enlarged (002) diffraction peaks of Cr*_x_* Ga_1−_ *_x_* Te exhibit a rightshift with the increased Cr concentration (right panel of Figure 1e), which is attributed to the decrease of the interlayer distance from 0.737 to 0.718 nm (2*d*sin*θ* = *λ*, where Cu K*α* radiation wavelength = 0.154 nm). Further, Figure 1f exhibits a typical Raman spectra of the as‐grown bulk pure GaTe and eight peaks, all belonging to the vibrational modes of monoclinic GaTe, consistent with the previous reports.^[^ ^24^, ^26^ ^]^ For the bulk Cr*_x_* Ga_1−_ *_x_* Te, modes at 116 cm^−1^ (A_g_) and 164 cm^−1^ (B_g_) are slight left‐shifted with increasing the Cr concentration (Figure 1g), as summarized in Figure 1h, which could be attributed to the local strain caused by the Cr substitution.^[^ ^27^ ^]^ Also, for a 16 nm thin nanosheet, due to the thickness significantly reduced compared with the bulk crystal, some Raman peaks from lattice vibrations are very different from bulk crystals for the quantum size or confinement effects (Figure S2, Supporting Information). For example, the 114 cm^−1^ (A_g_) and 162 cm^−1^ (B_g_) peaks for the 16 nm thin Cr_0.024_Ga_0.976_Te nanosheet are degraded due to the suppression of out‐of‐plane lattice vibration. Also, the 154 cm^−1^ (A_g_) and 169 cm^−1^ (A_u_) peaks emerge due to the enhancement of in‐plane lattice vibration.^[^ ^26^ ^]^

**Figure 2:**
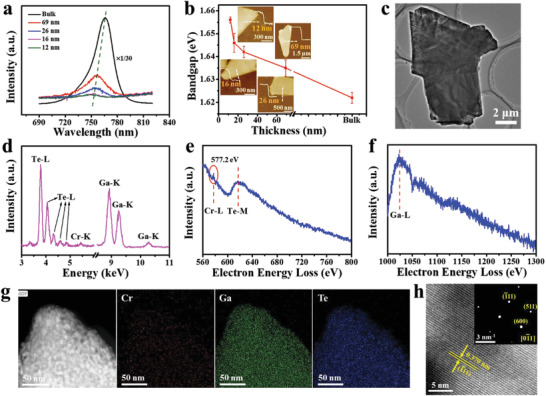
PL and TEM characterizations of vdW Cr_0.024_Ga_0.976_Te nanosheets. a) PL spectra of the Cr_0.024_Ga_0.976_Te with different thicknesses exfoliated on SiO_2_/Si by Scotch tape. The green dashed line guides the blue shift of the peak. b) Thickness‐dependent bandgap for the Cr_0.024_Ga_0.976_Te. Inset: AFM images of the four as‐tested Cr_0.024_Ga_0.976_Te nanosheets and the corresponding thickness. Error bars SD; *N* = 3. c) TEM image of a Cr_0.024_Ga_0.976_Te nanosheet on carbon millipore filter with good sheet orientation control. d) EDS spectra of the Cr_0.024_Ga_0.976_Te nanosheet. e,f) EELS of the Cr_0.024_Ga_0.976_Te nanosheets with identified Cr–L, Te–M, and Ga–L peaks. g) Cr, Ga, and Te element mapping of the Cr_0.024_Ga_0.976_Te nanosheet. Scale bar: 50 nm. h) HRTEM image of a monoclinic Cr_0.024_Ga_0.976_Te nanosheet. Upper right inset: SAED pattern from the same region.

To identify the semiconducting character of the Cr*_x_* Ga_1−_ *_x_* Te, we addressed electrical and optical measurements. For the electrical measurement, temperature‐dependent resistivities (*R*–*T*) of bulk GaTe and Cr*_x_* Ga_1−x_Te single crystals are all decreasing with rising temperature, suggesting the typical semiconducting behavior (Figure 1k, see details in the Experimental Section). The photoluminescence (PL) spectra of bulk GaTe and Cr*_x_* Ga_1−_ *_x_* Te crystals with different Cr concentrations are shown in Figure 1i, and the peak location red‐shifts with increased Cr concentration, resulting in the slight decrease of bandgap from ≈1.659 to ≈1.622 eV (Figure 1j). Additionally, for Cr_0.024_Ga_0.976_Te with reduced thickness, the PL peaks gradually move toward the short wavelength (**Figure** **2**a), which is due to the increase of bandgap from ≈1.622 to ≈1.657 eV (Figure 2b) and exhibits obvious 2D quantum confinement effect.^[^ ^28^, ^29^ ^]^ The Raman results in Figure S2 (Supporting Information) are consistent with these PL performance in thin Cr_0.024_Ga_0.976_Te nanosheet where blue shift of PL is very clear compared with the bulk crystals for the thickness reduction. Also, the PL mapping image of a 98 nm Cr_0.024_Ga_0.976_Te nanosheet presents the emission intensity mainly around 1120–1460 a.u. at 760 nm (Figure S3a,b, Supporting Information), demonstrating the homogeneous photoluminescence performance.

In order to investigate the crystal structure and element distribution of GaTe and Cr*_x_* Ga_1−_ *_x_* Te nanosheets, the transmission electron microscope (TEM) was performed on the exfoliated nanosheets. A TEM image and the corresponding EDS mapping of the GaTe nanosheet exhibit good orientation control and uniform element distribution (Figure S4a–c, Supporting Information). The atomic percent of Ga and Te are 50.27% and 49.73%, very close to the ratio of 1:1 (Figure S4f, Supporting Information). High‐resolution TEM (HRTEM) image of the same GaTe nanosheet reveals clear crystal lattices with two obvious planes’ spacing of 0.186 and 0.208 nm, matching well with ($7\bar{1}\bar{5}$) and ($51\bar{4}$) planes of the monoclinic GaTe (Figure S4d, Supporting Information). The interplanar spacing of ($11\bar{1}$) in GaTe was measured to be 0.375 nm from fast Fourier transformation (FFT) (Figure S4e, Supporting Information). For Cr_0.024_Ga_0.976_Te, a well‐oriented Cr_0.024_Ga_0.976_Te nanosheet on a copper grid and the corresponding EDS spectrum are exhibited in Figure 2c,d. Furthermore, Figure 2g presents the homogeneous distribution throughout the entire investigated region of Cr, Ga, and Te elements. The HRTEM image of the Cr_0.024_Ga_0.976_Te nanosheet shows clear lattice fringe and the interplanar spacing of ($\bar{1}11$) was measured to be 0.370 nm (Figure 2h). Meanwhile, the corresponding selected area electron diffraction (SAED) pattern reveals a typical single monoclinic phase. These results imply that the presence of Cr does not destroy the structural integrity of the GaTe single crystal matrix. Moreover, the electron energy loss spectroscopy (EELS) was applied to determine the valence of Cr in the Cr*_x_* Ga_1−_ *_x_* Te (Figure 2e,f).^[^ ^30^, ^31^ ^]^ According to the previous report,^[^ ^31^ ^]^ the Cr–L peak position is around 576.9–577.8 eV for Cr^2+^ and around 578.1–580.5 eV for Cr^3+^. As shown in Figure 2e, a Cr–L peak appears at 577.2 eV, suggesting the existence of Cr^2+^ in the Cr*_x_* Ga_1−_ *_x_* Te.

The ferromagnetism in vdW Cr*_x_* Ga_1−_ *_x_* Te bulk crystals was measured by the vibrating sample magnetometer (VSM) at different temperatures and magnetic fields. For comparison, the magnetic properties of the GaTe were measured first and show linear negative slope diamagnetic feature (Figure S5a,b, Supporting Information). However, obvious ferromagnetism is observed in Cr*_x_* Ga_1−_ *_x_* Te. Figure S6a–c (Supporting Information) exhibits the spontaneous magnetization without external magnetic field (*M*–*T* curves at 0 T, see details in the Experimental Section), zero‐field cooling (ZFC), and field cooling (FC) (*M*–*T* curves at 0.1 T) of the Cr*_x_* Ga_1−_ *_x_* Te crystals, indicating typical *T*_C_ are ≈326.9–328.9 K, demonstrating the intrinsic room‐temperature ferromagnetism in Cr*_x_* Ga_1−_ *_x_* Te crystals. By increasing the Cr content from 0.6% to 2.4%, the *M*_sat_ can be effectively adjusted ≈3.5 times at 3 K (0.73–2.52 emu g^−1^) and ≈5.4 times at 300 K (0.24–1.29 emu g^−1^) (Table S2, Supporting Information). Cr*_x_* Ga_1−_ *_x_* Te crystals show soft ferromagnetic behavior with the discernible coercivity (*H*_C_) 368.85–396.74 Oe (3 K) and 199.5–202.18 Oe (300 K), echoing the room‐temperature ferromagnetism in the Cr*_x_* Ga_1−_ *_x_* Te crystals (Figure S6d–f and Table S2, Supporting Information). Moreover, the *M*_sat_ and *H*_C_ of the Cr*_x_* Ga_1−_ *_x_* Te crystals tend to decrease with rising temperature, which indicates that the ferromagnetic phase is gradually transforming into the paramagnetic phase (Figure S7a,b, Supporting Information).

**Figure 3:**
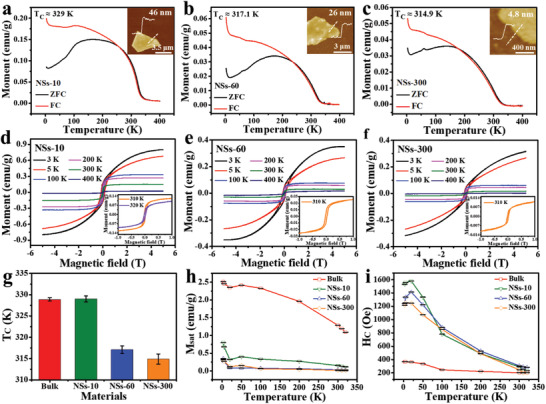
Ferromagnetic properties of the Cr_0.024_Ga_0.976_Te nanosheets with different thicknesses. a–c) *M*–*T* (ZFC–FC) curves of the Cr_0.024_Ga_0.976_Te NSs‐10 (a), NSs‐60 (b), and NSs‐300 (c) at 0.1 T external magnetic field. Insets: representative AFM images for NSs‐10, NSs‐60, and NSs‐300. d–f) *M*–*H* curves for the Cr_0.024_Ga_0.976_Te NSs‐10 (d), NSs‐60 (e), and NSs‐300 (f) in the magnetic field range from −5 to 5 T under different temperatures. Insets: slightly below *T*_C_ *M*–*H* hysteresis loops ranging from −1 to 1 T. g) *T*_C_ comparison of the Cr_0.024_Ga_0.976_Te bulk crystals, NSs‐10, NSs‐60, and NSs‐300. Error bars SD; *N* = 3. h,i) Temperature dependence of *M*_sat_ (h) and *H*_C_ (i) for the Cr_0.024_Ga_0.976_Te bulk crystals, NSs‐10, NSs‐60, and NSs‐300. Error bars SD; *N* = 200.

The vdW Cr*_x_* Ga_1−_ *_x_* Te single crystals can be transformed to thin nanosheets (NSs) with different thicknesses down to few layers (see details in the Experimental Section). Typically, the liquid exfoliation yields of the Cr_0.024_Ga_0.976_Te crystals after 10 min (NSs‐10), 60 min (NSs‐60), and 300 min (NSs‐300) ultrasonication were calculated as 6.82%, 8.90%, and 10.04%, respectively (Table S3, Supporting Information). The thicknesses of Cr_0.024_Ga_0.976_Te NSs‐10, NSs‐60, and NSs‐300 nanosheets have remarkable differences and were narrowly distributed mostly at ≈40 nm (≈40 layers), ≈24 nm (≈24 layers), and ≈9.5 nm (≈9 layers, 3–9 layers over 50%), respectively (Figures S8–S10 and more discussions in Note S1 in the Supporting Information). Before the VSM test, a millipore filter was used to collect the nanosheets with good orientation for their thin 2D sheet nature and the influence of the background signals from millipore filter was excluded (Figure S11, Supporting Information). According to the *M*–*T* curves under spontaneous magnetization regime (0 T, Figures S8c, S9h, and S10c, Supporting Information) and ZFC–FC regime (0.1 T, **Figure** **3**a–c) of the Cr_0.024_Ga_0.976_Te NSs‐10, NSs‐60, and NSs‐300, the *T*_C_ are recognized as ≈329, ≈317.1, and ≈314.9 K, respectively. The slight drop of *T*_C_ from bulk to thin nanosheet suggests typical thickness effects in Cr_0.024_Ga_0.976_Te, which also can be found in other vdW ferromagnetic crystals, indicating the influence of dimension and thermal fluctuation in 2D van der Waals crystals.^[^ ^11^, ^12^ ^]^ Moreover, *M*–*H* curves of the Cr_0.024_Ga_0.976_Te NSs‐10, NSs‐60, and NSs‐300 all exhibit the obvious ferromagnetic behavior at 300 K (Figure 3d–f) and the *M*_sat_ can be highly regulated ≈8.1 times at 3 K (2.52–0.31 emu g^−1^) and ≈75.9 times at 300 K (1.29–0.017 emu g^−1^) by decreasing the thickness from bulk to thin nanosheets (Table S3, Supporting Information). Figure 3g summarizes the *T*_C_ of the Cr_0.024_Ga_0.976_Te with different thicknesses, exhibiting the robust room‐temperature ferromagnetism in the Cr_0.024_Ga_0.976_Te thin nanosheets. The larger suppression of *M*_sat_ in the Cr_0.024_Ga_0.976_Te thin nanosheets at 300 K (Figure 3h) is attributed to the large influence of thermal fluctuations in the 2D ferromagnetic system^[^ ^11^ ^]^ and the reduction of *T*_C_.^[^ ^32^ ^]^ Similar with other vdW ferromagnetic crystals,^[^ ^33^, ^34^, ^35^ ^]^ the *H*_C_ of Cr_0.024_Ga_0.976_Te nanosheets exhibit an enhancement up to ≈4.1 times than bulk counterpart at 3 K, as shown in Figure 3i (and Table S2, Supporting Information). Furthermore, the magnetizations for both the Cr_0.024_Ga_0.976_Te bulk and the ultrathin nanosheets (NSs‐300) show in‐plane easy magnetization direction at 300 K (Figure S12a,b, Supporting Information).

**Figure 4:**
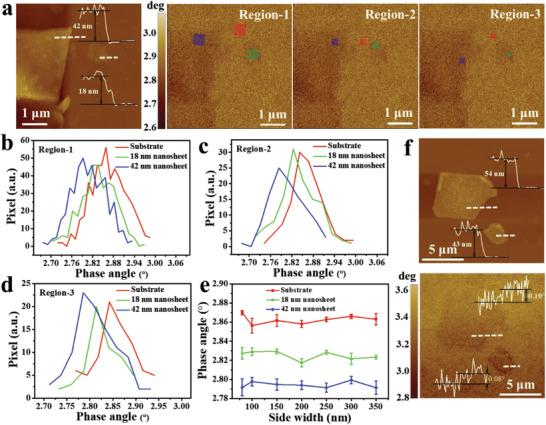
Room‐temperature ferromagnetism in single Cr_0.024_Ga_0.976_Te nanosheet by MFM. a) AFM topography (left) and the corresponding MFM phase images (right 3 pictures) of two Cr_0.024_Ga_0.976_Te single nanosheets with 42 and 18 nm thicknesses. b–d) MFM phase angle comparisons of the SiO_2_/Si substrate (red box), 18 nm single nanosheet (green box), and 42 nm single nanosheet (blue box) in (a) at three different square regions of different side widths. e) MFM phase angle of the SiO_2_/Si substrate, 18 nm nanosheet, and 42 nm nanosheet with the side width from 80 to 350 nm. Error bars SD; *N* = 3. f) AFM topography (up) and the corresponding MFM phase images (down) of two Cr_0.024_Ga_0.976_Te single nanosheets with 54 and 43 nm thicknesses. Inset profiles in AFM image show the thickness and inset profiles in MFM image show phase angle difference between nanosheet and substrate. More details about thickness dependence of phase angle difference in Figure S14 (Supporting Information).

The room‐temperature ferromagnetism in single Cr_0.024_Ga_0.976_Te nanosheet of different thicknesses was confirmed by the room‐temperature magnetic force microscopy (MFM) without external magnetic field (*B* = 0). To avoid interference from short‐range atomic forces, the Co–Al tip without pre‐magnetization was first applied to confirm this preclusion in the MFM mode (Figure S13 and more discussions in Note S2 in the Supporting Information). Subsequently, the topography and in situ MFM phase images of the 42 and 18 nm Cr_0.024_Ga_0.976_Te nanosheets were obtained by using a pre‐magnetized Co–Al tip (**Figure** **4**a). Uniform MFM signals were observed in both two Cr_0.024_Ga_0.976_Te nanosheets, which present the single‐sheet‐level room‐temperature 2D ferromagnetism. Further, Figure 4b–d compares the MFM phase angle signals of the substrate (SiO_2_/Si) and two nanosheets at three different squares of different side widths and selected locations. Slight changes of ferromagnetism were captured due to the high ferromagnetic signal sensibility of MFM phase. The MFM phase angle for the substrate, 18 nm nanosheet, and 42 nm nanosheet can be distinguished to even the side width of the selected regions down to 80 nm (Figure 4e). Additionally, the MFM phase angle differences between Cr_0.024_Ga_0.976_Te single nanosheets and SiO_2_/Si substrates are highly related with the thickness of single Cr_0.024_Ga_0.976_Te nanosheets, as shown in Figure 4f (and Figure S14, Supporting Information).

In general, ferromagnetism in the Cr–Te system is highly dependent on the valence state of Cr.^[^ ^36^ ^]^ With lower valence states, the higher *T*_C_ is usually observed in Cr–Te‐based system.^[^ ^36^, ^37^ ^]^ Therefore, the room‐temperature ferromagnetism in the Cr*_x_* Ga_1−_ *_x_* Te may originate from the Cr^2+^ in the Cr–Te system. To gain more insight into the nature of the ferromagnetism in the Cr*_x_* Ga_1−_ *_x_* Te, the spin‐resolved density of states (DOSs) and magnetic exchange have been studied by the density‐functional‐theory (DFT)‐based first‐principle calculations (Figures S15–S17, Supporting Information). The calculated spin‐resolved DOSs of the GaTe are symmetrical as expected, while they are asymmetrical in all the Cr*_x_* Ga_1−_ *_x_* Te crystals, which is responsible for and consistent with the observed ferromagnetism feature in the bulk and thin Cr*_x_* Ga_1−_ *_x_* Te nanosheets. Spin‐polarized states appear with the presence of Cr and increase with the Cr concentration (Figure S17, Supporting Information). Meanwhile, the theoretical calculations indicate that both the bulk and the 2D Cr*_x_* Ga_1−_ *_x_* Te with different Cr concentrations are semiconductors, consistent with the electrical and optical measurements above. The further analysis of the DOSs suggests that the magnetic moments of the Cr*_x_* Ga_1−_ *_x_* Te are mainly contributed by the Cr‐3d electrons (Figure S17, Supporting Information), and each Cr atom carries increased average magnetic moment with the increasing Cr concentration (Figure S15, Supporting Information).

## 3 Conclusion

Compared with other intrinsically ferromagnetic vdW crystals reported so far, Cr*_x_* Ga_1−_ *_x_* Te shows above‐room‐temperature *T*_C_ and is a powerful candidate for room‐temperature 2D spintronics. Considering the extremely low *T*_C_ (<70 K) of most reported intrinsically ferromagnetic vdW semiconductors (Table S4, Supporting Information), Cr_0.024_Ga_0.976_Te has a much higher *T*_C_ up to 328.9 K in the bulk (≈4.8 times of *T*_C_ for Cr_2_Ge_2_Te_6_^[^ ^11^ ^]^ and ≈5.4 times of *T*_C_ for CrI_3_^[^ ^9^ ^]^) and up to 314.9 K in the ultrathin nanosheets. Furthermore, Cr*_x_* Ga_1−_ *_x_* Te possesses a more moderate bandgap than other intrinsic vdW ferromagnetic semiconductors, providing the greater potential for carrier control. Such robust and highly tunable room‐temperature 2D ferromagnetism in vdW semiconductors offers a feasible strategy to realize room‐temperature electric‐field control ferromagnetism, 2D ferromagnetic heterostructures, and low‐power spintronic devices.

## 4 Experimental Section

### Growth of the GaTe and Cr*_x_* Ga_1−_ *_x_* Te Crystals

The GaTe and Cr*_x_* Ga_1−_ *_x_* Te bulk crystals were grown by a two‐step KCl‐flux method in evacuated quartz tubes. First, the high purity Ga sticks (Aladdin, 99.9999%) and Te powders (Aladdin, 99.99%) were weighed out in stoichiometric molar ratios of 1:1 and then sealed in an evacuated quartz ampoule at a base pressure of 6 × 10^−5^ mbar. Then, the ampoule was heated to 1223 K for 24 h solid reaction followed by slow cooling to 1043 K at the rate of 2 K h^−1^. Second, the obtained GaTe precursors were ground and fully mixed with the high purity Cr powders (Adamas, 99.95%) and Te powders (Aladdin, 99.99%) by using the agate mortar. This mixture was evacuated and sealed in a new quartz ampoule with the KCl powders (Aladdin, 99.99%), which acted as the flux. The stoichiometric molar ratios of these raw materials were Cr:GaTe:Te:KCl equal to 1:9:1:40, 2:8:2:40, and 3:7:3:40, respectively. Then, the sealed ampoule was set in the same heating process to 1223 K as first step to melt all of reactants followed by the Cr*_x_* Ga_1−_ *_x_* Te crystallization. After the reaction, the product was immersed in deionized water for 2 h and washed several times to remove the residual KCl completely.

### Cr*_x_* Ga_1−_ *_x_* Te Crystal Exfoliation

The thin Cr_0.024_Ga_0.976_Te nanosheets were obtained by the liquid exfoliation with sonication^[^ ^38^ ^]^ or mechanical exfoliation by the Scotch tape. For liquid exfoliation, three copies of 40 mg Cr_0.024_Ga_0.976_Te crystals were weighed and put into three glass bottles both with ethanol, respectively. Then, the dispersion solutions were sonicated for 10, 60, and 300 min, and then hold for 2, 10, and 16 h without stirring, respectively. The supernatants of the dispersion from 10, 60, and 300 min sonication were collected to obtain thin Cr_0.024_Ga_0.976_Te nanosheets with different thickness distributions, noted as NSs‐10, NSs‐60, and NSs‐300, respectively.

### Crystal Characterizations

The phase, morphology, components, and crystal structures of GaTe and Cr*_x_* Ga_1−_ *_x_* Te samples were performed by optical microscope (MV6100), powder XRD (Smartlab SE, Rigaku Corporation), X‐ray fluorescence microprobe (EDAX Inc., EAGLE III), field emission transmission electron microscope (FTEM, Talos F200X, FEI), and 300 kV Titan TEM (Titan G2 60‐300, FEI), respectively.The thickness was measured by the atomic force microscope (AFM, XE7, Park). The Raman and photoluminescence spectroscope (LabRAM HR800, Horiba JobinYvon) were obtained with an excitation laser of 532 nm. All the above tests were carried out at room temperature.

### Electrical Transport Measurements

The temperature dependence of resistivity (*R*–*T*) curves were measured in a physical property measurement system (PPMS, DynaCool, Quantum Design, USA) with the four‐terminal configuration. 25 times were conducted at each temperature point for average with a constant current mode. The cooling rate was set as 2 K min^−1^ with the same interval of 1 K.

### Magnetization Measurements

The magnetic properties were measured by a PPMS (DynaCool, Quantum Design, USA) equipped with a VSM. For the VSM sample preparation with good orientations, a Cr_0.024_Ga_0.976_Te solution was dropped onto a polyvinylidene fluoride or carbon millipore filter for realizing good sheet orientation control, as shown in Figure 2c. The nanosheets were oriented easily parallel to a millipore filter substrate due to their 2D sheet structures after natural drying. To determine the mass of collected Cr_0.024_Ga_0.976_Te NSs‐10, NSs‐60, and NSs‐300, the millipore filter was weighed before and after Cr_0.024_Ga_0.976_Te nanosheet deposition. To gain highly oriented Cr_0.024_Ga_0.976_Te nanosheets, the amount of the deposited Cr_0.024_Ga_0.976_Te nanosheets was controlled in range of 1–3 mg (weight sensitivity of electronic balance, 0.01 mg). Then, the millipore substrate was fixed parallel to the VSM quartz sample holder by a polytetrafluoroethylene (PTFE) microfilm winding. It is worth noting that the diamagnetic signals of millipore substrate and PTFE microfilm were included in the background signal test (Figure S11, Supporting Information) and could be ignored in subsequent VSM test. Except the magnetic anisotropy measurement in Figure S12 (Supporting Information), all of the magnetization measurements were carried out with the magnetic field in‐plane oriented because of the in‐plane easy magnetization. Measurements of bulk Cr_0.024_Ga_0.976_Te crystal were carried out under the same condition as Cr_0.024_Ga_0.976_Te nanosheets. To test the sample with spontaneous magnetization regime (*B* = 0), the samples were first heated to 400 K (above the Curie temperature *T*_C_) and hold for 5 min. Then, the magnetic field was raised to 2 T and decreased to 0 with oscillated mode to remove the remanence of the samples and the superconducting coil. After that, the temperature was reduced to 3 K under zero field (*B* = 0). During the cooling process, the magnetic moment of the sample was continuously measured by VSM with a vibration frequency of 40 Hz and amplitude of 2 mm. For all *M*–*T* and *M*–*H* curves, 200 times per point were recorded for average and the rate of change magnetic field was set as 100 Oe s^−1^ with the same interval of 500 Oe. The rate of temperature change was set as 2 K min^−1^.

### Magnetic Force Microscopy

Single nanosheet magnetic properties’ measurement was presented by AFM (XE7, Park) equipped with a magnetic Co–Al tip. In MFM mode, the pre‐magnetized Co–Al tip was raised 50 nm from its original height and scan rate was set 0.3 Hz to eliminate the interference of short‐range atomic forces. Imaging with the non‐pre‐magnetized tip was applied as control to further confirm the elimination of short‐range atomic forces (Figure S13, Supporting Information). All MFM tests were performed at room temperature without external magnetic field.

### First Principle Calculations

DFT calculations were performed using the Vienna ab Initio Simulation Package.^[^ ^39^ ^]^ The electron–ion interactions were described with the projector augmented wave method.^[^ ^40^ ^]^ For the exchange and correlation energy, the hybrid Heyd–Scuseria–Ernzerhof 06 functional^[^ ^41^ ^]^ was employed. To account for the dispersion vdW interactions in layered system, a semiempirical potential was introduced in the total energy functional (DFT‐D2).^[^ ^42^ ^]^ The cutoff energy for the plane‐wave basis was set to be 400 eV and the Brillouin zone was sampled 5 × 5 × 1 *k*‐point grid mesh. One or two Ga atoms were replaced by Cr dopant in a 2 × 3 × 1 supercell with 72 atoms to simulate doping. Relaxations were performed until force on each atom became less than 0.01 eV Å^−1^, and the criterion for the total energy was set as 10^−6^ eV. A vacuum space of 15 Å was constructed vertical to the layer to avoid interaction between adjacent periodic layers.

### Statistical Analysis

1) The intensities of XRD data in Figure 1e, Raman data in Figure 1g and Figure S2 (Supporting Information), and PL data in Figure 1i were normalized. 2) The error bars in Figures 1, 2, 3, 4 and Figures S7 and S14 (Supporting Information) were presented by mean ± standard deviation (SD). 3) The sample sizes (*N*) for Figures 1, 2, 3g, 4e and Figure S14 (Supporting Information) were 3. The sample sizes (*N*) for Figure 3h,i and Figure S7 (Supporting Information) were 200. 4) Microsoft Office Excel and Origin were used for statistical analysis.

## Conflict of Interest

The authors declare no conflict of interest.

## Supporting information

Supporting Information

## Data Availability

The data that support the findings of this study are available in the Supporting Information of this article.
