# Supplementary material for: Highly‐Tunable Intrinsic Room‐Temperature Ferromagnetism in 2D van der Waals Semiconductor Cr x Ga1− x Te
Source: Adv Sci (Weinh). 2021 Oct 27;9(1):2103173. doi: 10.1002/advs.202103173 (PMC8728846; doi:10.1002/advs.202103173)
Supplement: Supplementary file 1 — Supporting Information [file ADVS-9-2103173-s001.pdf]

© 2021 Wiley-VCH GmbH

ADVANCED SCIENCE
Open Access

Supporting Information

for *Adv. Sci.,* DOI: 10.1002/advs.202103173

Highly-Tunable Intrinsic Room-Temperature Ferromagnetism in Two-Dimensional van der Waals Semiconductor $Cr_xGa_{1-x}Te$

Gaojie Zhang[1,3,†], *Hao Wu*[1,3,†], *Liang Zhang*[2,†], *Shanfei Zhang*[1,3], *Li Yang*[1,3], *Pengfei Gao*[1,3], *Xiaokun Wen*[1,3], *Wen Jin*[1,3], *Fei Guo*[2], *Yuanmiao Xie*[2], *Hongda Li*[2], *Boran Tao*[2], *Wenfeng Zhang*[1,3], *Haixin Chang*[1,3,*]

[1]Center for Joining and Electronic Packaging, State Key Laboratory of Material Processing and Die & Mold Technology, School of Materials Science and Engineering, Huazhong University of Science and Technology, Wuhan 430074, China.

[2]Center for Materials Science and Engineering, School of Electrical and Information Engineering, Guangxi University of Science and Technology, Liuzhou 545006, China.

[3]Institute for Quantum Science and Engineering, Huazhong University of Science and Technology, Wuhan 430074, China.

[*]Corresponding author. E-mail: hxchang@hust.edu.cn

[†]These authors contribute equally to this work.

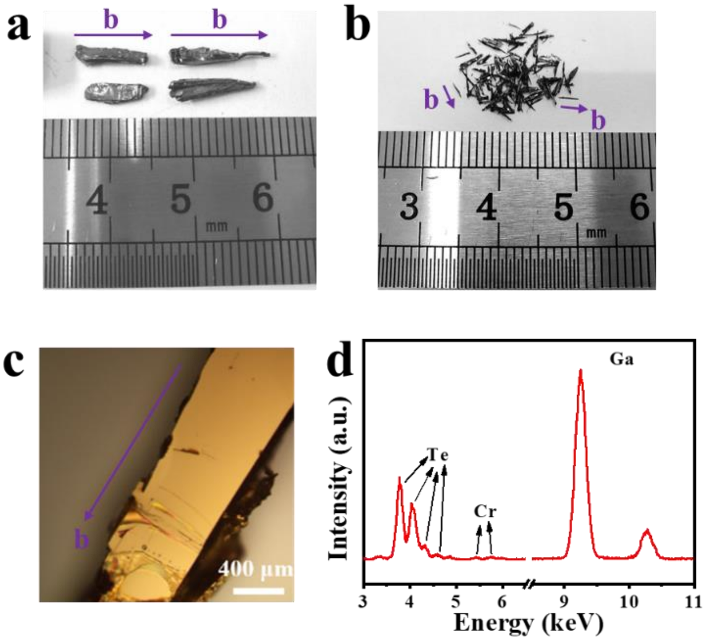

Figure S1. Optical images and XRF spectra of as-grown bulk GaTe and $Cr_xGa_{1-x}Te$ single crystals. (a) GaTe single crystals. (b) $Cr_xGa_{1-x}Te$ single crystals. (c) Optical image of a typical as-grown $Cr_xGa_{1-x}Te$ single crystal. Essentially, the striplike crystals have a longer crystal edge along the *b* axis (Purple arrows in each picture). (d) XRF spectra of the $Cr_{0.024}Ga_{0.976}Te$ single crystal in (c).

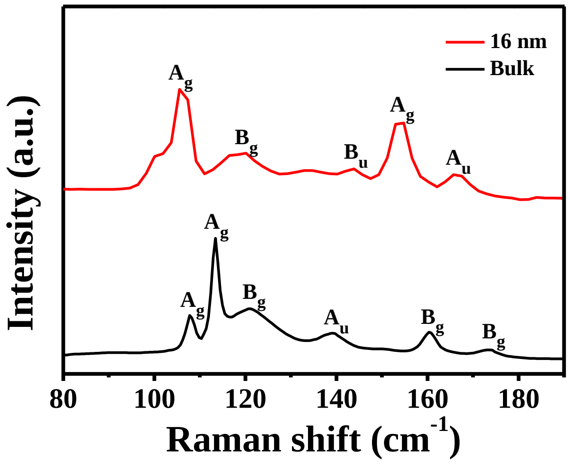

Figure S2. Raman spectra of the bulk $Cr_{0.024}Ga_{0.976}Te$ crystals and 16 nm $Cr_{0.024}Ga_{0.976}Te$ nanosheet.

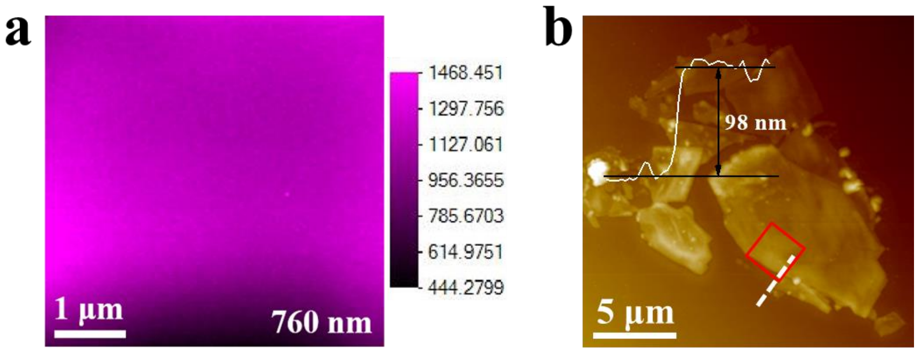

Figure S3. PL mapping and AFM test of a $Cr_{0.024}Ga_{0.976}Te$ nanosheet. (a) PL mapping of a $Cr_{0.024}Ga_{0.976}Te$ nanosheet at 760 nm wavelength. (b) AFM image of this as-tested $Cr_{0.024}Ga_{0.976}Te$ nanosheet and the red rectangle is the mapping region.

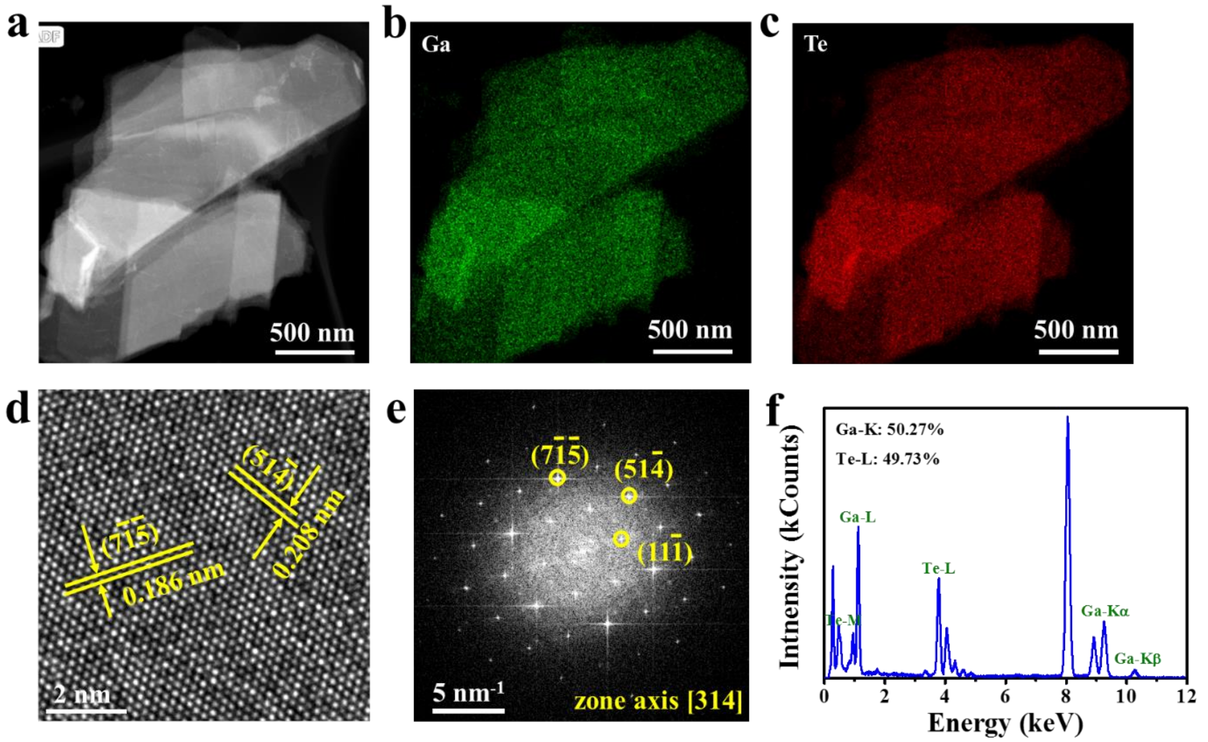

Figure S4. TEM characterizations of GaTe nanosheets. (a) STEM-HAADF image of GaTe nanosheets. Scale bar: 500 nm. (b, c) EDS mapping of Ga and Te elements in GaTe nanosheet, respectively. Scale bar: 500 nm. (d, e) HRTEM image and corresponding FFT pattern in (a). (f) EDS spectra and atomic percent of Ga and Te.

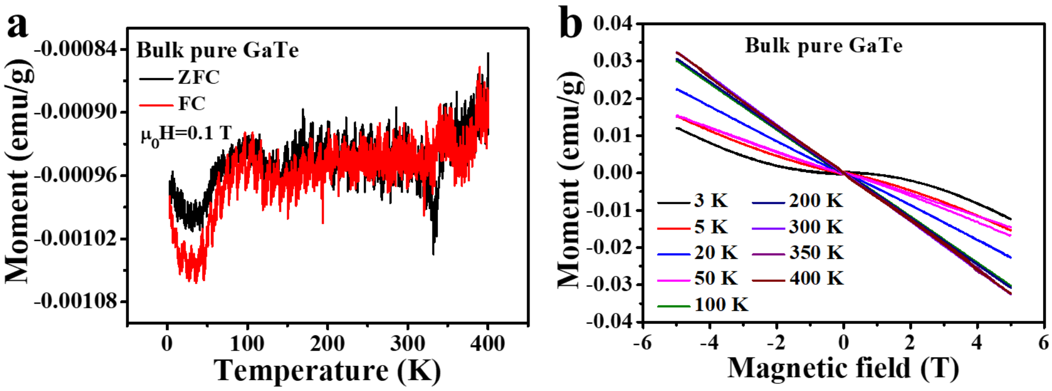

Figure S5. Magnetic properties of the GaTe crystals. (a) Temperature dependence of magnetic moment (M-T, ZFC-FC modes) curves of GaTe crystals at 0.1 T. (b) Magnetic field dependence of magnetic moment (M-H) curves for GaTe crystals at different temperatures ranging from 3 to 400 K.

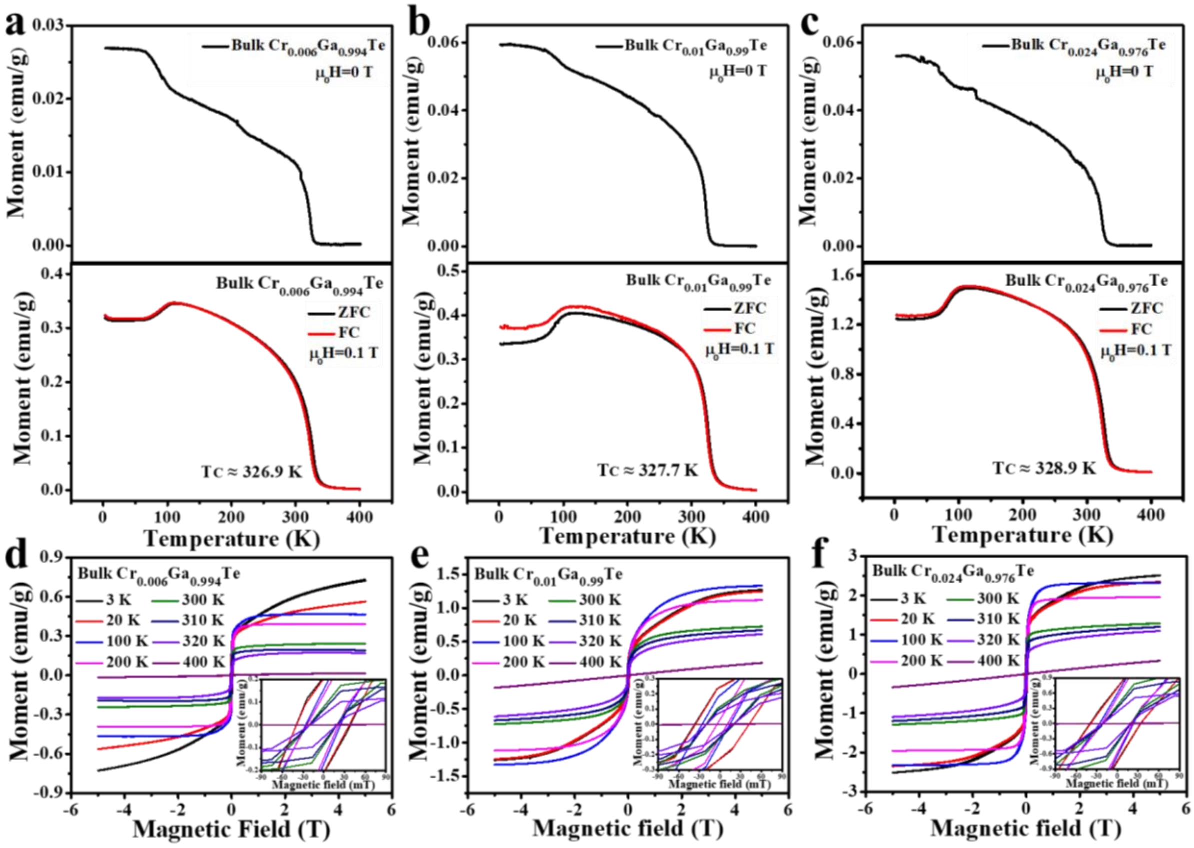

Figure S6. Ferromagnetic properties of the bulk $Cr_xGa_{1-x}Te$ crystals. (a-c) M-T curves of $Cr_xGa_{1-x}Te$ crystals from 3 to 400 K at 0 T (spontaneous magnetization regime) and 0.1 T (ZFC-FC regime) magnetic field. (d-f) M-H curves for $Cr_xGa_{1-x}Te$ crystals in the magnetic field range from -5 to 5 T under different temperatures. Insets: the corresponding partial enlarged images ranging from -90 to 90 mT.

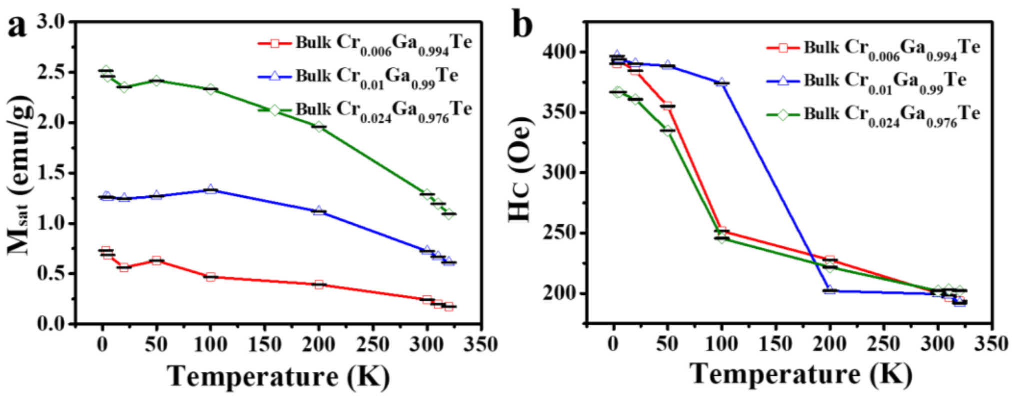

Figure S7. Temperature dependence of M_{sat} (a) and H_C (b) for $Cr_xGa_{1-x}Te$ crystals. Error bars SD; N=200.

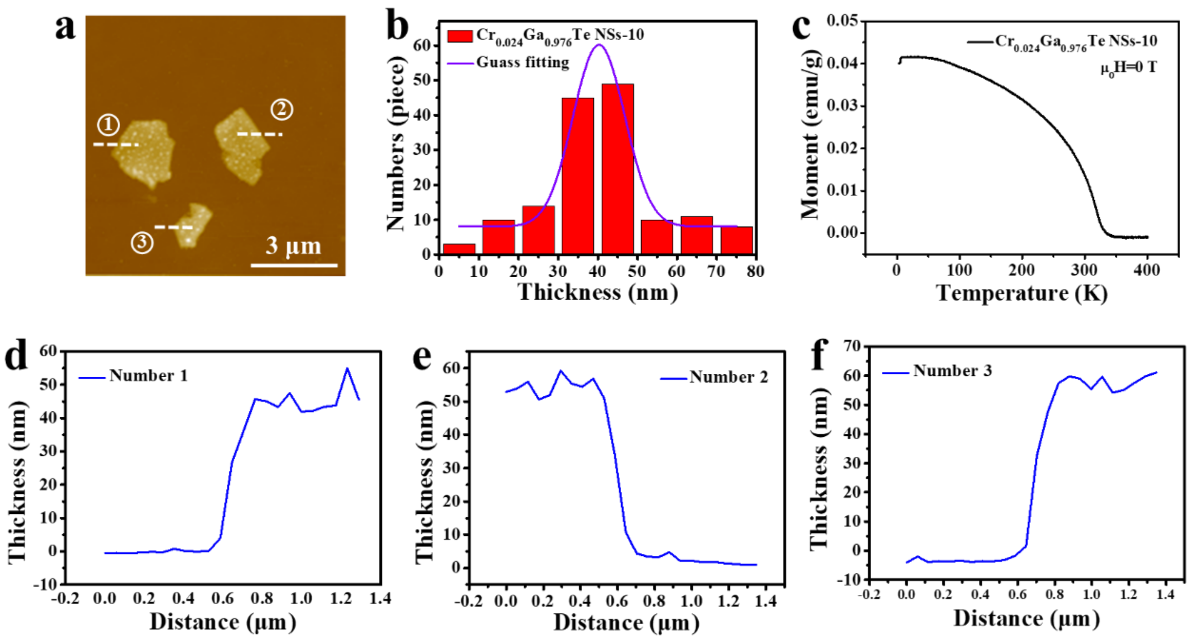

Figure S8. Thickness statistics of $Cr_{0.024}Ga_{0.976}Te$ NSs-10 (Sonicated 10 min and standing 2 h) nanosheets and spontaneous magnetization measurements without external magnetic field (B=0). (a, b) AFM images of three representative $Cr_{0.024}Ga_{0.976}Te$ NSs-10 nanosheets (a) and thickness distribution histograms (b) counted about 120~150 pieces nanosheets by AFM. (c) Spontaneous magnetization curves without external magnetic field (B=0) for $Cr_{0.024}Ga_{0.976}Te$ NSs-10. (d-f) Corresponding thickness of three $Cr_{0.024}Ga_{0.976}Te$ NSs-10 nanosheets along the dash line in (a).

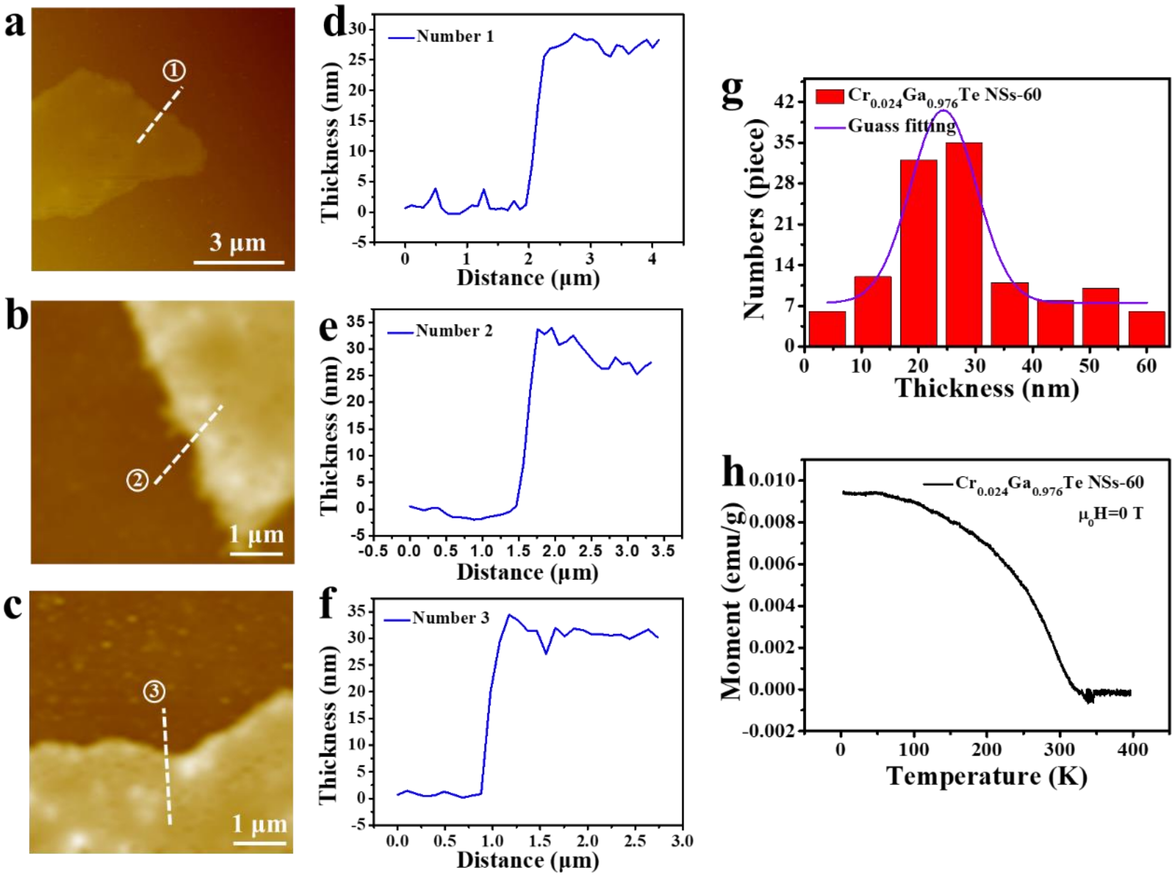

Figure S9. Thickness statistics of $Cr_{0.024}Ga_{0.976}Te$ NSs-60 (Sonicated 60 min and standing 10 h) nanosheets and spontaneous magnetization measurements without external magnetic field (B=0). (a-c) AFM images of a representative $Cr_{0.024}Ga_{0.976}Te$ NSs-60 nanosheet. (d-f) Corresponding thickness of a $Cr_{0.024}Ga_{0.976}Te$ NSs-60 nanosheet along the dash line in (a-c). (g) Thickness distribution histograms counted about 120~150 pieces nanosheets by AFM. (h) Spontaneous magnetization curves without external magnetic field (B=0) for $Cr_{0.024}Ga_{0.976}Te$ NSs-60.

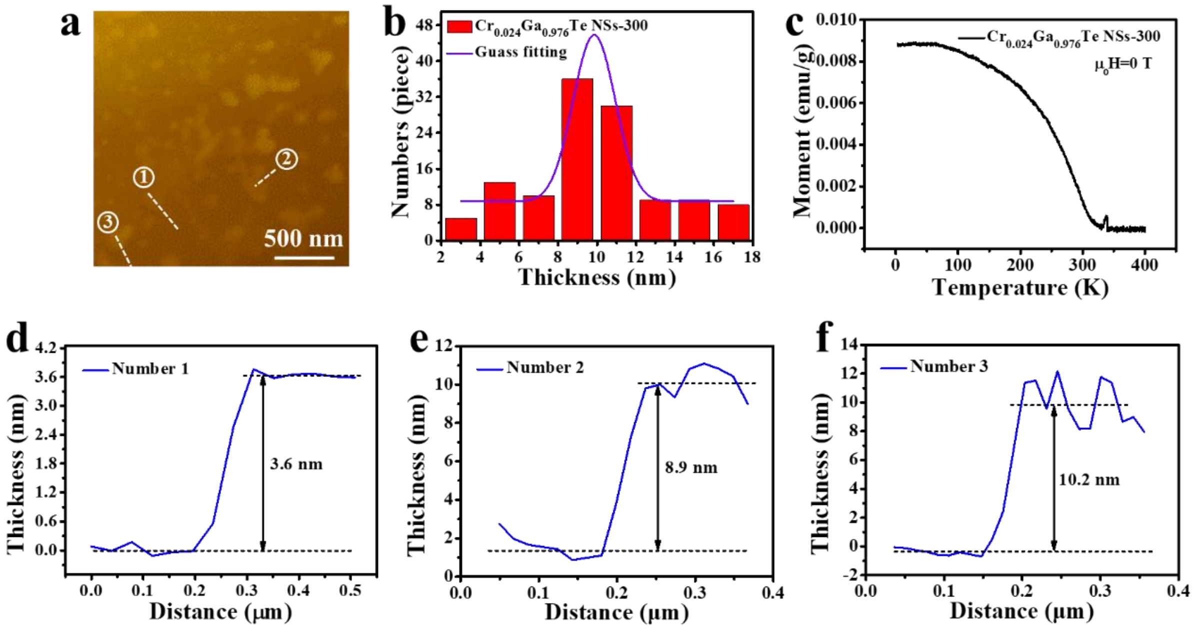

Figure S10. Thickness statistics of $Cr_{0.024}Ga_{0.976}Te$ NSs-300 (Sonicated 300 min and standing 16 h) nanosheets and spontaneous magnetization measurements without external magnetic field (B=0). (a, b) AFM images of some representative $Cr_{0.024}Ga_{0.976}Te$ NSs-300 nanosheets (a) and thickness distribution histograms (b) counted about 120~150 pieces nanosheets by AFM. (c) Spontaneous magnetization curves without external magnetic field (B=0) for $Cr_{0.024}Ga_{0.976}Te$ NSs-300. (d-f) Corresponding thickness of three $Cr_{0.024}Ga_{0.976}Te$ NSs-300 nanosheets along the dash line in (a).

Notes 1:

Due to the extremely weak magnetic moment of a single ferromagnetic nanosheet, we collected a large number of nanosheets through liquid exfoliation for direct magnetization test (**Figure S8-S10,** see details in **Experimental Section**). These nanosheets have good thickness control down to few layers by carefully adjusting the exfoliating and standing time. We counted about 120~150 pieces nanosheets by AFM and the thickness of NSs-10, NSs-60 and NSs-300 were mostly narrowly distributed ~40 nm (~40 layers), ~24 nm (~24 layers) and ~9.5 nm (~9 layers, 3-9 layers over 50%), respectively. Importantly, the spontaneous magnetization behaviors without external magnetic field (B=0) of these as-collected $Cr_{0.024}Ga_{0.976}Te$ nanosheets including NSs-300 prove the intrinsic room temperature ferromagnetism.

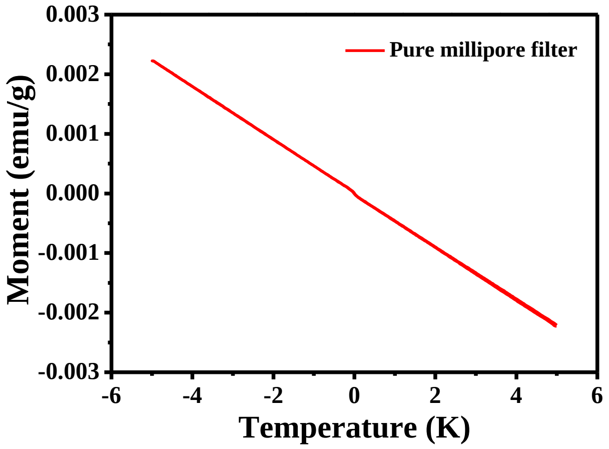

Figure S11. Diamagnetic property of pure millipore filter blank substrate at 300 K.

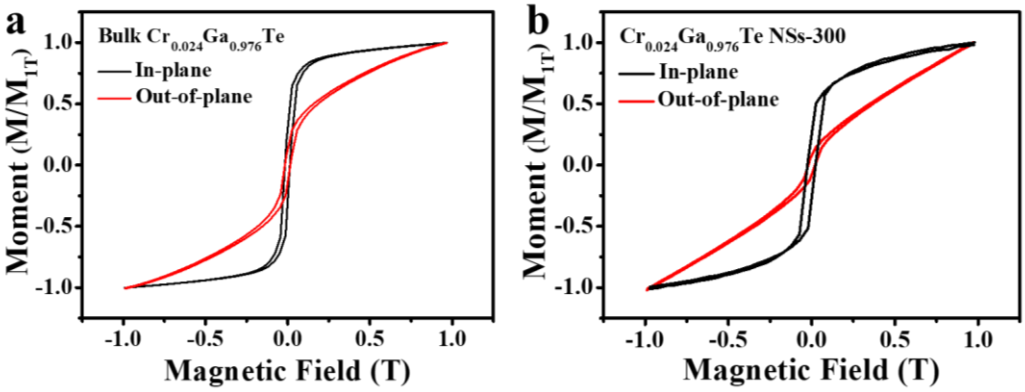

Figure S12. Magnetic anisotropy for $Cr_{0.024}Ga_{0.976}Te$ bulk crystals and ultrathin nanosheets at 300 K. (a) In-plane and out-of-plane VSM test of bulk $Cr_{0.024}Ga_{0.976}Te$. (b) In-plane and out-of-plane VSM test of $Cr_{0.024}Ga_{0.976}Te$ NSs-300.

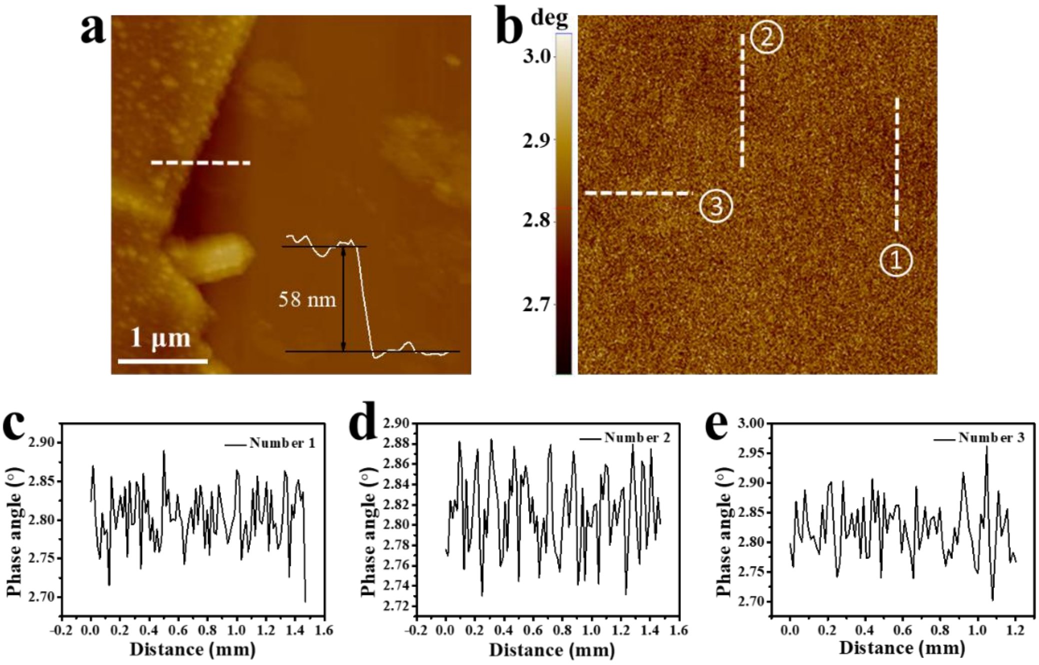

Figure S13. Room temperature MFM measurements for a 58 nm $Cr_{0.024}Ga_{0.976}Te$ nanosheet with non-pre-magnetized Co-Al tip. (a) AFM topography image. (b) Corresponding MFM phase image. (c-e) The change of phase angle along three dashed lines in (b). No external magnetic field was applied on this $Cr_{0.024}Ga_{0.976}Te$ nanosheet during the MFM tests.

Notes 2:

In general, the interference of atomic force to MFM phase angle will increase with the increase of sample thickness when keeping the same lifting height, scanning rate and sample type. To ensure the MFM phase signals that we obtained are from the long-range ferromagnetic force of $Cr_xGa_{1-x}Te$ rather than short-range atomic force, a non-pre-magnetized Co-Al tip was applied to scan the 58 nm $Cr_{0.024}Ga_{0.976}Te$ nanosheet with 50 nm lift and 0.3 Hz scan rate in MFM mode. Also, we carefully measured the phase angle changes of three different positions in the MFM image and presented the results in **Figure S13c-e**. Although this 58 nm controlled sample is the thickest one compared with other single nanosheets in **Figure 4,** its MFM signal from non-pre-magnetized tip is negligible compared with MFM signals of other thinner single nanosheets by pre-magnetized tip. Therefore, we believe the lift height and scan rate in our experiments are enough to avoid the interference of atomic force. The control blank test results here also demonstrate the MFM tests in **Figure 4** are reliable.

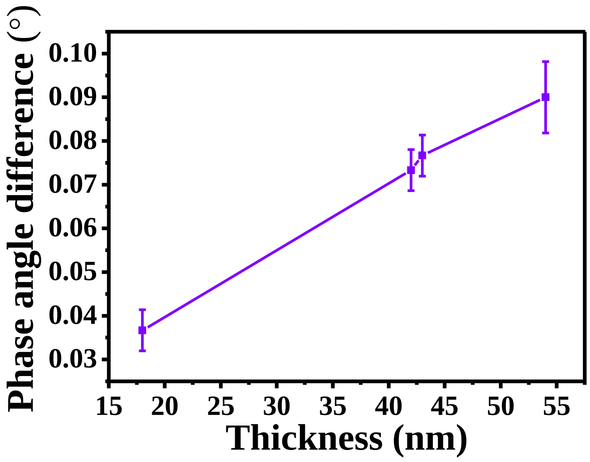

Figure S14. Thickness dependence of MFM phase angle difference between $Cr_{0.024}Ga_{0.976}Te$ nanosheet and SiO_2/Si substrate in MFM imaging. Error bars SD; N=3.

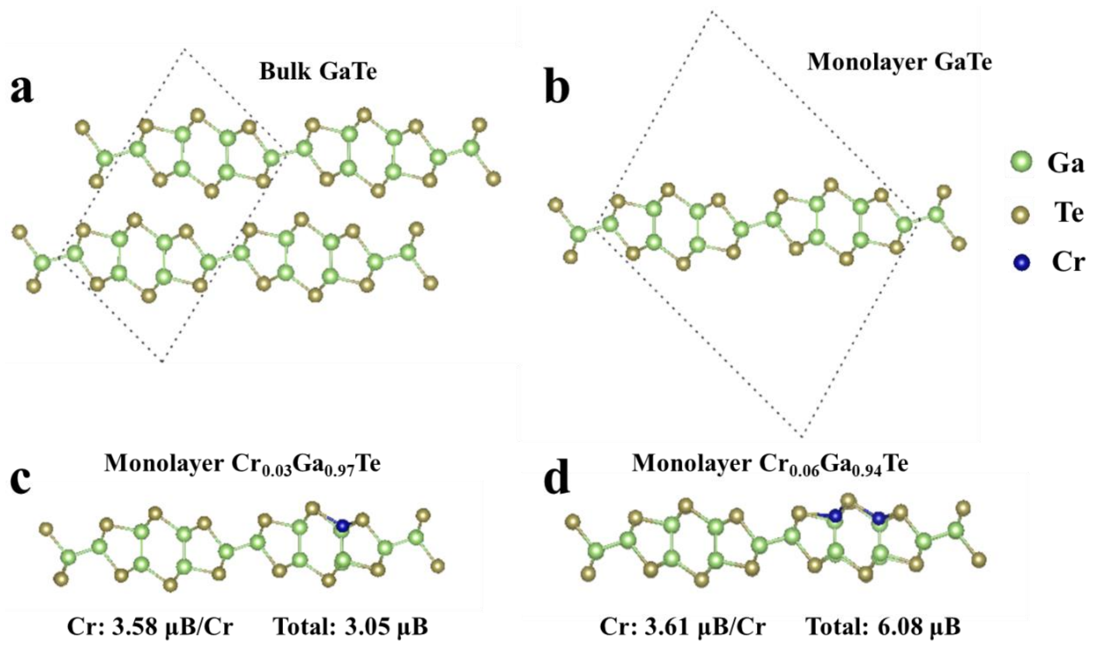

Figure S15. Crystal calculation models of GaTe, $Cr_{0.03}Ga_{0.97}Te$ and $Cr_{0.06}Ga_{0.94}Te$ crystals. (a, b) Crystal calculation models of bulk (a) and monolayer (b) GaTe crystals. (c, d) Crystal calculation models of monolayer $Cr_{0.03}Ga_{0.97}Te$ (c) and $Cr_{0.06}Ga_{0.94}Te$ (d) crystals. The magnetic moment of Cr and total are presented.

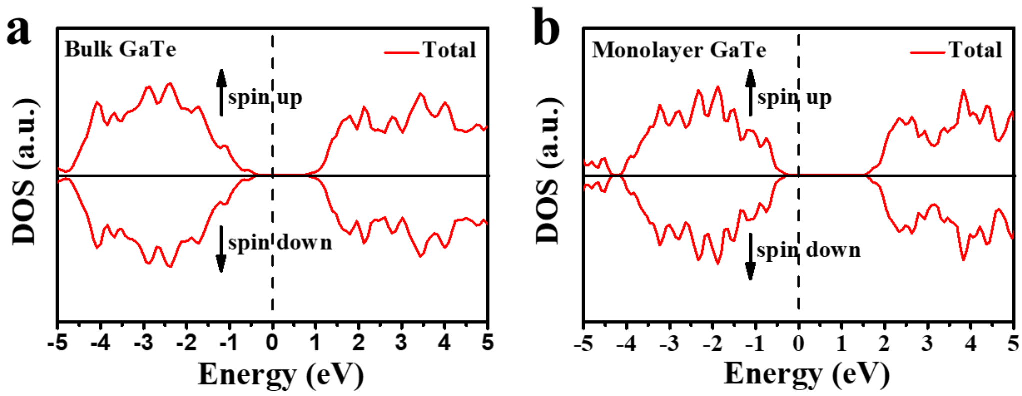

Figure S16. The spin-resolved density of states (DOSs) of bulk (a) and monolayer (b) GaTe crystals. The vertical dash lines denote the position of Fermi level.

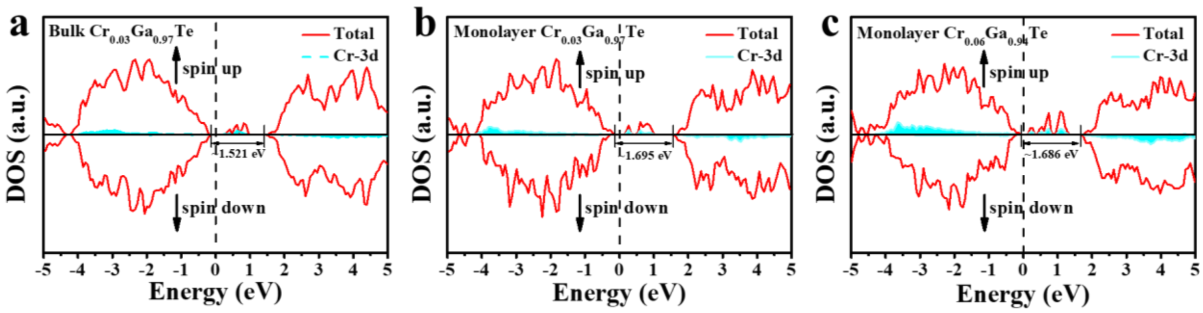

Figure S17. The spin-resolved density of states (DOSs) of bulk, monolayer $Cr_{0.03}Ga_{0.97}Te$ and monolayer $Cr_{0.06}Ga_{0.94}Te$ crystals. (a, b) Total DOS and partial DOS of Cr-3d states in bulk (a) and monolayer (b) $Cr_{0.03}Ga_{0.97}Te$ crystals. (c) Total DOS and partial DOS of Cr-3d states in monolayer $Cr_{0.06}Ga_{0.94}Te$ crystal. The vertical dash lines denote the position of Fermi level.

Table S1. The energy dispersive spectrum (EDS) analysis of three vdW $Cr_xGa_{1-x}Te$ thin crystals with different Cr concentration.

**Samples**	**Elements**	**Atomic percent (%)**
$Cr_{0.006}Ga_{0.994}Te$	Cr-K	0.31
	Ga-K	49.83
	Te-L	49.86
$Cr_{0.01}Ga_{0.99}Te$	Cr-K	0.49
	Ga-K	49.58
	Te-L	49.93
$Cr_{0.024}Ga_{0.976}Te$	Cr-K	1.2
	Ga-K	48.85
	Te-L	49.95

Notes 3:

The Cr atomic concentration (x) in $Cr_xGa_{1-x}Te$, is ~0.62%, ~0.98% and ~2.4%, which is marked as the value of 0.6%, 1% and 2.4%. This result indicates the successful incorporation of Cr into GaTe lattices.

Table S2. Comparison of saturation magnetic moment and coercivity for $Cr_xGa_{1-x}Te$ bulk crystals and $Cr_{0.024}Ga_{0.976}Te$ thin nanosheets measured at 3 K and 300 K.

**Materials**	**Moment (emu/g)**		**Coercivity (Oe)**	
	**3K**	**300K**	**3K**	**300K**
$Cr_{0.006}Ga_{0.994}Te$ bulk	0.73	0.24	392.77	200.34
$Cr_{0.01}Ga_{0.99}Te$ bulk	1.26	0.72	396.74	199.50
$Cr_{0.024}Ga_{0.976}Te$ bulk	2.52	1.29	368.85	202.18
$Cr_{0.024}Ga_{0.976}Te$ NSs-10	0.80	0.15	1531.36	289.40
$Cr_{0.024}Ga_{0.976}Te$ NSs-60	0.35	0.03	1241.64	305.81
$Cr_{0.024}Ga_{0.976}Te$ NSs-300	0.31	0.017	1216.91	246.18

Table S3. Exfoliation yield analysis of 10 min (2 h standing), 60 min (10 h standing) and 300 min (16 h standing) ultrasonication crystals. In order to obtain the liquid exfoliation yield of $Cr_{0.024}Ga_{0.976}Te$ crystals, we first weighed the initial crystals mass and total mass of crystals and bottle, respectively. After mixing the crystals with ethanol, ultrasound and standing for a period of time, we extracted all the supernatant and dried the precipitate in vacuum. Finally, we weighted the residual mass of crystals and bottle. The liquid exfoliation yield is calculated as

$$\text{Exfoliation yield} = \frac{M_{TM}\text{-}M_{RM}}{M_{IM}} \times 100\%$$

where M_{TM} is total mass of crystals and bottle, M_{RM} is residual mass of crystals and bottle, M_{IM} is initial crystals mass.

	**10 min**	**60 min**	**300 min**
Initial crystals mass (g)	0.01056	0.01056	0.01056
Total mass of crystals and bottle (g)	4.86921	4.89635	4.98833
Volume of ethanol (ml)	3	3	3
Residual mass of crystals and bottle (g)	4.86849	4.89541	4.98727
Exfoliation yield (%)	6.82	8.90	10.04

Table S4. Comparison of $Cr_{0.024}Ga_{0.976}Te$ and other vdW type experimentally realized ferromagnetic semiconductors.

Materials	Dimension	Bandgap (eV)	T_C (K)	H_C (Oe)	Ref
$CrBr_3$	Bulk	0.57±0.04	-	-	[1]
		1.35	37	-	[2]
		-	33	-	[3]
		1.68	-	-	[4]
		2.1	-	-	[5]
	Monolayer	-	34	-	[2]
CrI_3	Bulk	-	61	-	[6]
		1.2	68	-	[7]
	Monolayer	-	45	500@15 K	[6]
		1.1	-	550@15 K	[8]
$Cr_2Ge_2Te_6$	Bulk	0.74	61(±1)	＜100@-	[9]
		0.7	66	-	[10]
		-	68	-	[11]
	Bilayer	-	30	-	[11]
$CrSiTe_3$	Bulk	0.4	33	20@2 K	[12]
		-	33.2	-	[13]
VI_3	Bulk	0.6	49	10000@2 K	[14]
		0.67	50	9100@2 K	[15]
Fe-doped SnS_2	Monolayer	-	31	400@2 K	[16]
Nb, Co-doped WSe_2	Bulk	-	10	1200@10 K	[17]
$Cr_{0.024}Ga_{0.976}Te$	Bulk	1.622	328.9	368.85@3 K	**This work**
	NSs-10	1.635 (69 nm)	329	1531.36@3 K	
	NSs-60	1.642 (26 nm)	317.1	1241.64@3 K	
	NSs-300	1.656 (12 nm)	314.9	1216.91@3 K	

References

[1] D. Baral, Z. Fu, A. S. Zadorozhnyi, R. Dulal, A. Wang, N. Shrestha, U. Erugu, J. Tang, Y. Dahnovsky, J. Tian, T. Chien, *Phys Chem Chem Phys* **2021**, 23, 3225.

[2] Z. Zhang, J. Shang, C. Jiang, A. Rasmita, W. Gao, T. Yu, *Nano Lett.* **2019**, 19, 3138.

[3] X. Yu, X. Zhang, Q. Shi, S. Tian, H. Lei, K. Xu, H. Hosono, *Front Phys-Beijing* **2019**, 14, 43501.

[4] J. F. Dillon, H. Kamimura, J. P. Remeika, *J. Appl. Phys.* **1963**, 34, 1240.

[5] K. K. KANAZAWA, G. B. STREE, *Phys. Status Solidi* **1970**, 38, 445.

[6] B. Huang, G. Clark, E. Navarro-Moratalla, D. R. Klein, R. Cheng, K. L. Seyler, D. Zhong, E. Schmidgall, M. A. McGuire, D. H. Cobden, *Nature* **2017**, 546, 270.

[7] J. F. Dillon, C. E. Olson, *J. Appl. Phys.* **1965**, 36, 1259.

[8] K. L. Seyler, D. Zhong, D. R. Klein, S. Gao, X. Zhang, B. Huang, E. Navarro-Moratalla, L. Yang, D. H. Cobden, M. A. McGuire, W. Yao, D. Xiao, P. Jarillo-Herrero, X. Xu, *Nat. Phys.* **2017**, 14, 277.

[9] H. Ji, R. A. Stokes, L. D. Alegria, E. C. Blomberg, M. A. Tanatar, A. Reijnders, L. M. Schoop, T. Liang, R. Prozorov, K. S. Burch, N. P. Ong, J. R. Petta, R. J. Cava, *J. Appl. Phys.* **2013**, 114, 114907.

[10] Y. Liu, C. Petrovic, *Physical Review B* **2017**, 96, 054406.

[11] C. Gong, L. Li, Z. Li, H. Ji, A. Stern, Y. Xia, T. Cao, W. Bao, C. Wang, Y. Wang, *Nature* **2017**, 546, 265.

[12] L. D. Casto, A. J. Clune, M. O. Yokosuk, J. L. Musfeldt, T. J. Williams, H. L. Zhuang, M. W. Lin, K. Xiao, R. G. Hennig, B. C. Sales, J. Q. Yan, D. Mandrus, *APL Mater.* **2015**, 3, 041515.

[13] T. J. Williams, A. A. Aczel, M. D. Lumsden, S. E. Nagler, M. B. Stone, J. Q. Yan, D. Mandrus, *Phys. Rev. B* **2015**, 92, 144404.

[14] T. Kong, K. Stolze, E. I. Timmons, J. Tao, D. Ni, S. Guo, Z. Yang, R. Prozorov, R. J. Cava, *Adv. Mater* **2019**, 31, 1808074.

[15] S. Son, M. J. Coak, N. Lee, J. Kim, T. Y. Kim, H. Hamidov, H. Cho, C. Liu, D. M. Jarvis, P. A. C. Brown, J. H. Kim, C.-H. Park, D. I. Khomskii, S. S. Saxena, J.-G. Park, *Phys. Rev. B*

2019, 99, 041402.

[16] B. Li, T. Xing, M. Zhong, L. Huang, N. Lei, J. Zhang, J. Li, Z. Wei, *Nat. Commun.* **2017**, 8, 1.

[17] S. Ahmed, X. Ding, P. P. Murmu, N. Bao, R. Liu, J. Kennedy, L. Wang, J. Ding, T. Wu, A. Vinu, *Small* **2020**, 16, 1903173.
